# Temporal dynamic of cognitive decline in type 2 diabetes mellitus patients: a multimodal biomarker analysis using event-based modal and principal component analysis

**DOI:** 10.1186/s13098-025-02003-0

**Published:** 2025-11-14

**Authors:** Min-Hua Ni, Bo Hu, Xiao-Yan Bai, Yao Tong, Zi-Yang Ma, Hao Xie, Xin-Yu Cao, Yan-Yan Cui, Si-Ning Li, Pan Dai, Li-Juan Du, Xin-Wen Yu, Lin-Feng Yan, Bin Gao, Ying Yu, Guang-Bin Cui

**Affiliations:** 1 Department of Radiology, Tangdu Hospital, Fourth Military Medical University, 569 Xinsi Road, Xi’an, 710038 Shaanxi Province China; 2 Department of Endocrinology, Tangdu Hospital, Fourth Military Medical University, 569 Xinsi Road, Xi’an, 710038 Shaanxi China

**Keywords:** Cognitive decline, Event-based model, Gray matter volume, Principal component analysis, Type 2 diabetes mellitus

## Abstract

**Background:**

Type 2 diabetes mellitus (T2DM) is an important risk factor for cognitive impairment. Prior research has shown cognitive deficits and neural alterations across multiple domains in T2DM patients. However, the sequential dynamics of cognitive decline in this population remain poorly understood. This study employs an integrative approach combining Principal Component Analysis (PCA) and the Event-Based Model (EBM) to identify the temporal sequence of cognitive changes and underlying neural mechanisms in T2DM.

**Methods:**

This study assessed 119 T2DM patients and 87 healthy controls with neuropsychological tests and Magnetic Resonance Imaging for gray matter volume (GMV). PCA was used to reduce dimensionality in CVLT, STROOP, and WCST due to their substantial number of items, enabling integration into the EBM model. EBM estimated the sequence of cognitive and neurostructural changes. Partial correlation analyses were used to examine associations with clinical factors with controlling covariance.

**Results:**

Cognitive decline in T2DM began with attention and working memory, followed by executive function and episodic memory. GMV loss started in the insular gyrus, spreading to other regions. T2DM patients exhibited significantly more advanced disease progression than healthy controls (EBM stage 0.54 (0.12) vs. 0.49 (0.10), *P* = 0.001). A negative correlation linked long-delay memory (CVLT-PC4) to random blood glucose (*r* = -0.581, *P*_FDR_ = 0.025).

**Conclusion:**

Memory decline and insular gyrus atrophy may serve as early biomarkers for T2DM-related cognitive impairment. These findings highlight potential targets for early intervention and suggest strategies for developing personalized treatments to improve life quality in affected individuals.

**Supplementary Information:**

The online version contains supplementary material available at 10.1186/s13098-025-02003-0.

## Introduction

Diabetes mellitus is a complicated metabolic disorder characterized by hyperglycemia and insulin resistance, with type 2 diabetes mellitus (T2DM) accounting for 90–95% of cases [46, 47]. Cognitive impairment is one of the common complications of T2DM and leads to difficulties in self-management and social functions, significantly decreasing the quality of life [3, 64].

While substantial evidence has characterized the neurocognitive manifestations of T2DM as multidimensional manifestations including verbal and visual memory, attention and concentration, processing speed, executive function, and motor control, the developmental trajectories of these domain-specific deficits demonstrate non-linear progression patterns [1, 22, 46]. Additionally, neuroimaging advances have delineated morphometric alterations of cognitive impairment [12]. Prior research has demonstrated that T2DM patients with cognitive impairment exhibit reduced gray matter volume (GMV) in several brain regions, including the superior temporal gyrus, right middle frontal gyrus, right Rolandic operculum, left fusiform gyrus, cerebellum, and frontal opercular cortex [53, 61, 62]. Converging longitudinal and cross-sectional work further identifies the hippocampus as one of the earliest and most consistently affected structures in T2DM, with poorer glycaemic control being linked to smaller hippocampal volumes and episodic memory deficits [34, 37]. However, extant investigations manifest two critical methodological constraints: (1) the predominant reliance on cross-sectional designs obscures the temporal dynamics of cognitive deterioration and neuroanatomical trajectories of GMV reorganization in T2DM patients, hindering identification of critical windows for intervention; (2) multidimensional clinical assessments demonstrate substantial psychometric redundancy, necessitating dimensionality reduction to retain informative features for further exploration [4, 32].

Principal component analysis (PCA) is a dimensionality reduction technique that identifies latent variables by decomposing covariance structures through orthogonal transformation of high-dimensional datasets [26, 43]. The event-based model (EBM) estimates the ordered abnormality sequence of biomarkers by combining severity information across biomarkers and individuals, without reference to a given individual’s clinical status [18]. Previous studies have successfully applied these methods to various neurodegenerative diseases, including Alzheimer’s disease (AD) [18, 33], Parkinson’s disease [48], schizophrenia [28], and multiple sclerosis [17], indicating that this method is effective and robust. However, current methodological innovations remain conspicuously underutilized in T2DM research, particularly regarding the development of longitudinal profiling of cognitive decline and GMV trajectory.

In this study, we established an integrative analytical framework combining EBM with PCA to decode temporal sequences of cognitive deterioration and imaging biomarkers in T2DM using cross-sectional datasets. After multimodal integration, we systematically investigate clinical correlation between disease-specific event patterns and pathophysiological biomarkers in T2DM. The findings may offer evidentiary support in clinical decision-making, demonstrating potential clinical utility particularly regarding cognitive trajectory monitoring and identification of critical interventional windows.

## Materials and methods

### Participants

One hundred and nineteen T2DM patients and eighty-seven HC subjects were recruited from the endocrinology department of Tangdu hospital and the local community. The T2DM patients were defined as fasting blood glucose (FBG) ≥ 7.0 mmol/L and/or 2-hour post oral glucose tolerance test (OGTT) glucose ≥ 11.1 mmol/L. The subjects with FBG < 6.1 mmol/L and 2-hour post-OGTT glucose < 7.8 mmol/L were included in HC group. Participants were excluded if they had (i) other types of diabetes (type 1 diabetes or gestational diabetes); (ii) neurological disorders of the central nervous system or diseases seriously impairing neurological function; (iii) any psychiatric or neurological illness; (iv) retinopathy or neuropathy; (v) a history of substance, alcohol or drug abuse. To capture the full spectrum of cognitive performance across varying degrees of impairment, no clinical diagnosis of mild cognitive impairment or subjective cognitive decline was performed at enrolment, and cognitive performance was not used to include or exclude participants with T2DM. A brief, retrospective estimate of baseline cognitive profile based solely on MMSE and MoCA cut-offs is provided in Supplementary Methods for descriptive purposes only.

The clinical characteristics data included age, sex, years of education, body mass index (BMI), disease duration, blood pressure, FBG, Postprandial blood glucose (PBG), Random blood glucose (GLU), hemoglobin A1C (HbA1c), total cholesterol, triglyceride, high-density lipoprotein cholesterol (HDL-C), low-density lipoprotein cholesterol (LDL-C), and urinary microalbumin (MAlb).

Blood samples were collected after an overnight fast of ≥ 8 h (07:00–09:00); participants were asked to abstain from alcohol, caffeine and strenuous exercise for 24 h prior to the visit. A second tube for 2-h post-prandial glucose was drawn after a standard 75-g oral glucose load. All samples were centrifuged (3 000 rpm, 10 min, 4 °C) and analysed on the same day (Beckman Coulter AU 5800).

### Neuropsychological tests

All participants completed the MMSE, the MoCA, Self-Assessment Scale for Anxiety (SAS), Self-Rating Scale for Depression (SDS), the California Verbal Learning Test (CVLT), the Wisconsin Card Sorting Test (WCST), the Stroop Color-Word Test (STROOP), and Trail Making Test (TMT). This battery of psychological assessment was used to assess general cognitive capability, anxiety and depressive states, memory function, and executive function, respectively.

For all tests, higher values indicate better performance unless stated otherwise. TMT completion time (s) was recorded raw; longer times reflect poorer performance. CVLT: higher correct recall/hits and lower false-positive indicate better memory. Stroop: more correct items and smaller incongruent-baseline interference denote better executive control. WCST: more correct categories and fewer perseverative errors signify better cognitive flexibility.

### Data acquisition

MRIs were acquired using a 3.0 T GE Discovery MR 750 scanner (GE Healthcare, Milwaukee, WI, USA) with an eight-channel prototype quadrature birdcage head coil array. Foam padding was used to limit head motion. Throughout the scan, participants lay supine with eyes closed and were instructed to remain awake without engaging in deliberate thoughts. Structural images were acquired by using three-dimensional brain volume (3D-BRAVO) and the routine clinical protocol (T1 weighted images, T2 weighted images, T2 fluid attenuated inversion recovery images, and time-of-flight magnetic resonance angiography) were acquired to detect brain abnormalities. Detailed MRI settings were detailed in previous publications [25, 39].

### Data preprocessing

Three-dimensional T1-weighted images from all participants underwent preprocessing using Statistical Parametric Mapping version 12 (SPM12) and the Computational Anatomy Toolbox (CAT) 12.9 (r2577) in MATLAB R2023a. The preprocessing pipeline encompassed image segmentation, registration, and spatial normalization. For the segmentation process, the East Asian brain-specific tissue probability maps (TPMs) from the CAT12 were utilized. The imaging data underwent intrinsic resampling using a spatially adaptive non-local mean filter to 1.5 × 1.5 × 1.5 mm^3^, followed by bias field correction and affine registration to standard space. Subsequently, the images were processed through the standard SPM pipeline including “unified segmentation” algorithm and skull stripping procedures. This comprehensive preprocessing workflow ensured accurate tissue classification while maintaining anatomical consistency across subjects.

Then, tissue classification was refined through local intensity modulation across gray matter, white matter, and cerebrospinal fluid. Adaptive maximum a posteriori segmentation with partial volume estimation quantified fractional tissue composition at the voxel level, accompanied by total intracranial volume (TIV) quantification. Subsequently, spatial normalization to Montreal Neurological Institute (MNI) reference space was achieved through high-dimensional diffeomorphic registration using geodesic shooting algorithms [5]. All images with data quality below level C were excluded and finally spatial smoothing using an isotropic Gaussian kernel (6 mm full width at half maximum) to mitigate inter-subject anatomical variability while preserving mesoscopic structural features. For details on the CAT12 quality scoring system used to ensure image quality, please refer to the Supplementary Materials.

Here, all of the Anatomical Automatic Labeling (AAL) were separated into 17 features (frontal lobe, temporal lobe, parietal lobe, occipital lobe, insula, cingulate, sensorimotor, Broca’s area, cerebellum, hippocampus, parahippocampus, amygdala, caudate, putamen, pallidum, nucleus accumbens and thalamus) which were selected as the region of interests (ROIs), as details described in previous study [29].

### PCA

To reduce the scores of two cognitive domains (memory and executive function) to more meaningful components and meet the requirements of subsequent EBM model input data, PCA was applied to the obtained z-scores of the CVLT, STROOP and WCST for each subject. PCA is an efficient dimensionality reduction technique that simplifies data structures by transforming high-dimensional features into an orthogonal set of low-dimensional PCs. This approach significantly reduces the complexity of the data while preserving key information [52].

PCA was conducted on the cognitive domain scores using the scikit-learn library version 0.24 within a Python 3.9 environment. The selection of PCs was guided by the criteria established by Jolliffe [2], which recommends that the cumulative explained variance should exceed 70%, and that the eigenvalues of the components should be greater than 0.7, ensuring robust representation of data variability [31]. In this study, PCA was used to reduce the cognitive scores into two main components: memory and executive function. This reduction was necessary to meet the input requirements of the EBM model, which requires a manageable number of input features to effectively estimate the sequence of cognitive and neurostructural changes, providing a more interpretable and manageable dataset for subsequent analysis.

### EBM

EBM is a sophisticated data-driven model that forecasts disease progression by mapping the timeline of biomarker abnormalities using a single cross-sectional dataset [43, 58]. This methodology is particularly effective in cohorts with diverse disease severity, as early biomarkers tend to show abnormal values more frequently than those that manifest later in the disease’s course. The sequence of events is probabilistically determined in a data-driven fashion by aggregating the severity of biomarkers, which is indicative of their event probabilities, across multiple individuals. In essence, biomarkers with a higher prevalence are positioned earlier in the sequence. The model has been extensively utilized in research to delineate the progression and sequence of AD cognitive impairment characteristics [16, 18, 40]. Its strengths are manifold: (1) By capitalizing on the fact that included subjects exhibit varying degrees of cognitive impairment, we can deduce the sequence of disease characteristic changes during the dynamic evolution of cognitive impairment from cross-sectional study data. (2) The model employs a probabilistic approach that automatically discerns the normal and abnormal distributions of each input feature from the data, eliminating the need for manual outlier definition and thereby reducing the influence of subjective error. These features make EBM to be a robust tool for understanding the complex trajectories of cognitive impairment diseases [19].

#### Spatiotemporal cascades of cognitive function scores or GMV abnormalities

We used EBM to estimate the spatiotemporal cascade of abnormal biomarker events, which in this study refers to the three-step process of fitting the longitudinal order of different cognitive function dimensions scores and brain structural changes (GMV) in each T2DM patient. First, EBM estimates the degree of abnormality of each biomarker by linearly mapping the cognitive function scores or GMV of each subject to probabilities of regional abnormality (0: no abnormality, 1: mild abnormality (33%), 2: moderate abnormality (67%), 3: severe abnormality (100%)). This mapping allows us to quantify the severity of cognitive and neurostructural changes in a standardized manner. Second, EBM estimates the spatiotemporal cascade of events for each subject, by ordering these probabilities. Third, the mean spatiotemporal cascade for cognitive function scores or GMV is estimated as the sequence that minimizes the sum of probabilistic Kendall’s Tau distances to the spatiotemporal cascades of T2DM subjects [51]. In addition to determining the average sequence of biomarker abnormalities, the methodology also quantifies the relative temporal intervals separating these events. This analysis yields a collection of “event-centers” (ECs), which are positioned along a disease progression timeline that is normalized to span from 0 to 1. These ECs serve as reference points, illustrating the chronological order and relative timing of biomarker abnormalities within the context of disease development [50].

#### Patients staging

This study employs the Expectation-Maximization (EM) algorithm to optimize the disease staging for individual samples. By constructing a likelihood function that integrates the conditional probabilities of various biomarkers at different time points, the function assesses the likelihood of the dataset given a specific sequence of disease progression. The EM algorithm is utilized to iteratively identify the sequence that maximizes the likelihood function, thereby determining the most accurate stage of disease progression for the individual [58].

#### Validation

We conducted cross-validation on our event-based models by re-estimating each complete model, which includes event distributions and maximum likelihood sequences, using 100 bootstrap samples with replacement.

#### Statistical analysis

**Fig. 1:**
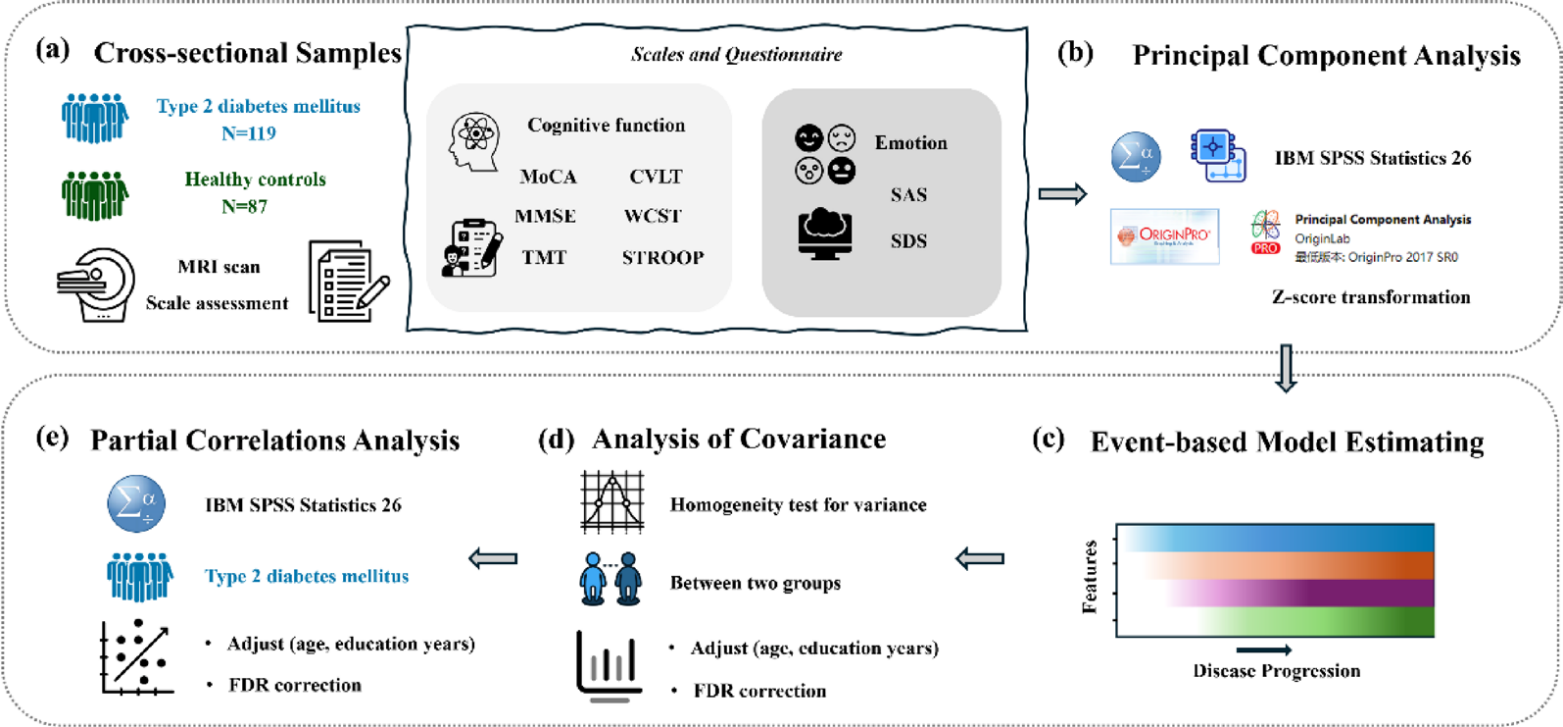
Flow diagram for experimental flow. MRI: Magnetic Resonance Imaging; MoCA: Montreal Cognitive Assessment; MMSE: Mini-Mental State Examination; CVLT: California Verbal Learning Test; STROOP: Stroop Color Word Test; WCST: Wisconsin Card Sorting Test; TMT: Trail Making Test; SAS: Self-Rating Anxiety Scale; SDS: Self-rating depression scale; FDR: false discovery rate

Statistical analyses were conducted using the Statistical Package for the Social Sciences (SPSS version 26.0). We calculated the mean (standard deviation [SD]) or median (interquartile range [IQR]) of characteristics for the entire study population. Multivariate analysis of covariance (ANCOVA) was used to compare differences in demographic, clinical characteristics, MoCA scores, MMSE scores, TMT-A, TMT-B, SAS scores, SDS scores, CVLT principal components (PCs), STROOP PCs, and WCST PCs between T2DM and the HC groups. The analysis was adjusted for baseline age, sex, and education level. Differences in disease progression stages estimated by EBM (EBM staging) were compared using a two-sample t-test, as covariates had already been adjusted in the EBM fitting. Partial correlation analysis was used to estimate the correlation between cognitive or GMV indicators that showed significant difference between the two groups and clinical risk factors, adjusting for baseline age, sex, and education level. The false discovery rate (FDR) method in MATLAB R2023b platform was used for the multiple comparison correction of the correlation analysis. Significant levels were set at *P* < 0.05 after FDR correction. Flow diagrams are shown in Fig. 1.

## Results

### Demographic and clinical characteristics of the participants

Demographic and clinical data were evaluated between the T2DM and HC groups (Table 1). After covariance analysis between these two groups, the results revealed that there were no significant differences in BMI, postprandial blood glucose, microalbuminuria, total cholesterol, triglycerides, HDL-C, LDL-C and diastolic pressure. Higher age (*P* < 0.001), HbA1c *(P* < 0.001), fast blood glucose *(P* < 0.001), random blood glucose *(P* < 0.001), systolic pressure (*P* = 0.021) and lower education years (*P* = 0.001) were found in the T2DM group.

**Table 1:** Demographic and clinical characteristics of the participants

	T2DM (*n* = 119)	HC (*n* = 87)	F/χ^2^	*P*
Age (years)	54.871 ± 9.074	49.922 ± 7.621	4.136	< 0.001^**^
Education (years)	12.020 ± 3.063	13.663 ± 3.888	−3.257	0.001^*^
Male/female	87/32	50/37	5.517	0.019^*^
Diabetes duration (years)	9.632 ± 7.037	–	–	–
BMI (kg/m^2^)	25.061 ± 3.290	24.126 ± 3.245	0.467	0.497
Biochemical indicator				
HbA1c (%)	8.575 ± 2.060	5.561 ± 0.297	15.630	< 0.001^**^
Fasting blood glucose (mmol/L)	7.942 ± 2.910	3.972 ± 2.436	10.628	< 0.001^**^
Postprandial blood glucose(mmol/L)	10.977 ± 4.186	7.320 ± 0.910	1.623	0.208
Random blood glucose (mmol/L)	8.503 ± 2.848	5.978 ± 1.025	6.253	< 0.001^**^
Microalbuminuria (mmol/L)	79.703 ± 151.511	17.420 ± 11.797	0.134	0.716
Total cholesterol (mmol/L)	4.388 ± 1.129	4.696 ± 1.077	−0.020	0.888
Triglycerides (mmol/L)	1.861 ± 1.275	1.937 ± 1.322	−1.634	0.207
HDL (mmol/L)	1.097 ± 0.240	1.196 ± 0.385	−0.030	0.864
LDL (mmol/L)	2.542 ± 1.294	2.658 ± 0.745	−0.058	0.811
Systolic pressure (mmHg)	130.216 ± 16.060	126.750 ± 18.450	5.631	0.021^*^
Diastolic pressure (mmHg)	81.897 ± 9.364	82.026 ± 12.666	−0.523	0.473

Data were reported as mean ± SD. T2DM, type 2 diabetes mellitus; HC, healthy controls; BMI: Body Mass Index; HbA1c: hemoglobin A1C; HDL: high-density lipoprotein; LDL: low-density lipoprotein. ^*^P < 0.05; ^**^ *P* < 0.001

### Cognitive characteristics of the participants

**Fig. 2:**
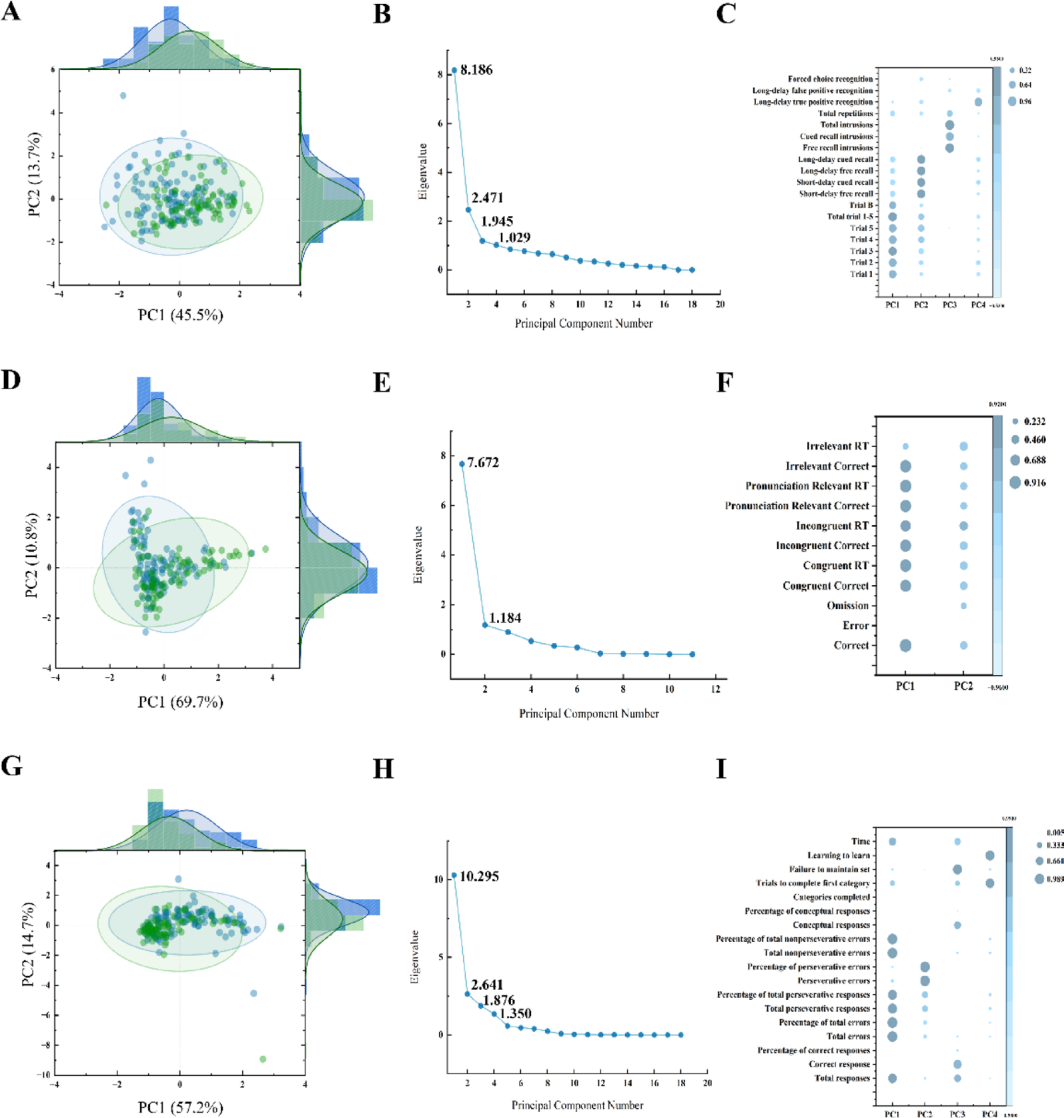
The principal component of multiple cognitive function estimated by the PCA.** (A)(D)(G)** Two-dimensional scatter plots of PCA for the CVLT, STROOP and WCST respectively, each plot showing the relationship between the two PC (PC1 and PC2). Each dot represents a sample, and the colors and shapes may represent different categories or groups. The ellipse represents the distribution of the data with a 95% confidence interval. **(B)(E)(H)** The lithotripsy map of PCA for the CVLT, STROOP and WCST respectively. Each plot shows the eigenvalues of the PC. The PC whose eigenvalue is greater than 1 is selected as the effective principal component. There were four effective components in CVLT, and the eigenvalues were 8.19, 2.47, 1.95 and 1.03, respectively. There were two effective components in STROOP, and the eigenvalues were 7.67 and 1.84, respectively. There were 4 effective components in WCST, and the eigenvalues were 10.30, 2.64, 1.88 and 1.35, respectively. **(C)(F)(I)** The bubble diagram of PCA for the CVLT, STROOP and WCST respectively. Each diagram shows the loadings of different variables on the PC. The load represents the degree to which each variable contributes to the PC. The points in the diagram represent the load value of each variable on different PCs, and the size of the points may represent the absolute value of the load. PCA: principal component analysis; CVLT: California Verbal Learning Test; STROOP: Stroop Color Word Test; WCST: Wisconsin Card Sorting Test; PC: principal component

Detailed information on the CVLT, STROOP and WCST assessment of the two groups is presented in Table **S1**. The Kaiser-Meyer-Olkin test indicated a measure of sampling adequacy of 0.708, 0.782, 0.735 respectively and all Bartlett’s test of sphericity were found to be significant (*P* < 0.001), suggesting that the CVLT, STROOP and WCST results were suitable for PCA. The rubble diagram of PCA is shown in Fig. 2. Gravel with eigenvalue greater than 1 is selected as the main component.

Four PCs were extracted from the CVLT accounting for 71.56% of the total variable. The first component (PC1) mainly reflected immediate recall explaining 45.48% of the total variables including the trial 1, trial 2, trial 3, trial 4, trial 5, trial 1–5 and trial B. The second component (PC2) mainly reflected long delayed recognition accounting for 13.73% of the total variance including the short-delay free recall, short-delay cued recall, long-delay free recall and long-delay cued recall. The third component (PC3) mainly reflected intrusion and repetition explaining 6.64% of the total variables including the free recall intrusions, cued recall intrusions, total intrusions and total repetitions. The fourth component (PC4) reflected long delayed recognition explaining 5.72% of the total variables including long-delay true positive recognition and long-delay false positive recognition. A synthesized score (CVLT-PC) was calculated based on the four above components, providing a comprehensive assessment of episodic memory.

Two PCs were extracted from the STROOP accounting for 80.51% of the total variable. The PC1 mainly reflected correct and reaction time explaining 69.75% of the total variables including the correct number, congruent correct number, congruent reaction time, incongruent number, incongruent reaction time, pronunciation relevant correct number, pronunciation relevant reaction time, irrelevant correct number and irrelevant reaction time. The PC2 mainly reflects error and omission accounting for 10.76% of the total variance including the error number and omission number. A synthesized score (STROOP-PC) was calculated based on the two above components, providing a comprehensive assessment of executive function including attention and inhibitory control.

As for WCST, four PCs were extracted from this score accounting for 89.80% of the total variable. The PC1 mainly reflected Overall cognitive function and cognitive flexibility explaining 57.22% of the total variables including the total response, total errors, percentage of total errors, total perseverative responses, percentage of total perseverative responses, total non-perseverative errors, percentage of total non-perseverative errors and time. The PC2 mainly reflects perseverative errors explaining 14.65% of the total variance including the perseverative errors and percentage of perseverative errors. The PC3 mainly reflected abstract thinking explaining 10.42% of the total variables including the total correct responses, the conceptual responses and failure to maintain set. The PC4 mainly reflected learning ability explaining 7.51% of the total variables including the complete first category and learning to learn. A synthesized score (WCST-PC) was calculated based on the four above components, providing a comprehensive assessment of executive function including cognitive flexibility, attention, and abstract thinking.

### Sequence of cognitive decline in the T2DM group

**Fig. 3:**
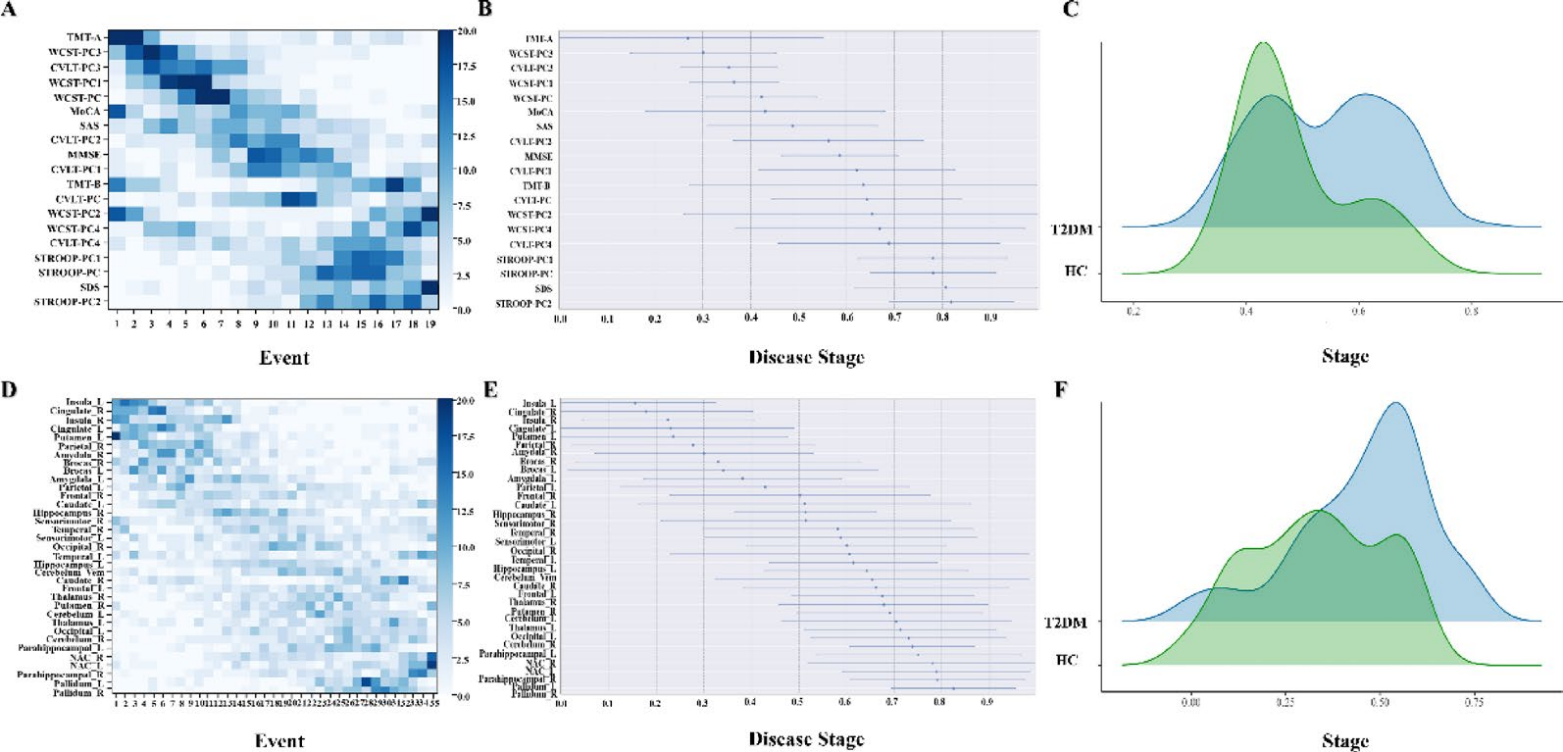
The sequence of multi-dimension cognitive function and brain imaging biomarkers estimated by the EBM.** (A)(D)** the saturation gradient of each square corresponds to the frequency of parameter localization during bootstrap resampling, with maximal chromatic density identifying the predominant temporal sequence for each biomarker in cognition and GMV in T2DM group respectively. **(B)(E)** the event center and variance diagram illustrates the estimated stage at which the features deviated from normality and the associated variance within the population in cognition and GMV in T2DM group respectively. **(C)(F)** the demographic distribution of all subjects at different EBM stages in cognition and GMV respectively. MoCA: Montreal Cognitive Assessment; MMSE: Mini-Mental State Examination; CVLT: California Verbal Learning Test; PC: principal component; STROOP: Stroop Color Word Test; WCST: Wisconsin Card Sorting Test; TMT: Trail Making Test; SAS: Self-Rating Anxiety Scale; SDS: Self-rating depression scale; L: left; R: right; NAC: Nucleus Accumbens; EBM: event-based model; T2DM: type 2 diabetes mellitus

The probability sequence of detectable cognitive changes in the T2DM group shown in Fig. 3 (A-B), which was estimated by the EBM method using bootstrapping, using a set of neuropsychological tests in our battery. The posterior position variance indicates the degree of confidence (from left to right) of the model with respect to ordering (from top to bottom) and the dark sections of positional variance show high confidence in the ordering.

**Fig. 4:**
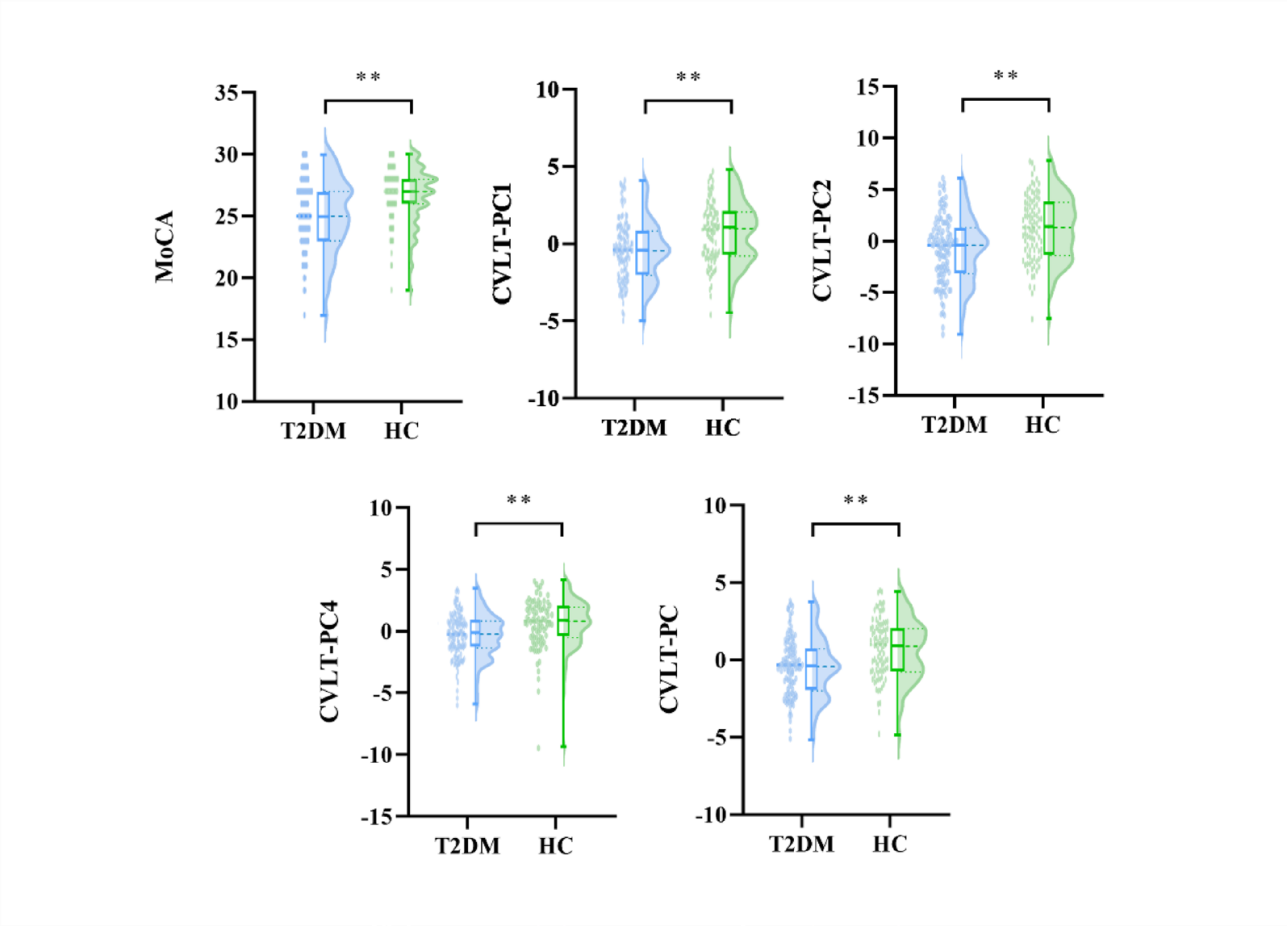
Between-group comparison of multi-dimension cognitive function and brain imaging biomarkers. MoCA: Montreal Cognitive Assessment; CVLT: California Verbal Learning Test; PC: principal component; T2DM: type 2 diabetes mellitus; HC: healthy controls; FDR: false discovery rate. ^**^ *P* < 0.05 after FDR correction

After applying PCA to reduce the dimensionality of the cognitive scale, the results from the EBM demonstrated that the TMT-A (a measure of attention and working memory) tends to show abnormalities earlier in the T2DM group, while the model estimates relatively late deficits in executive function followed by episodic memory, general cognition and general mental status. Along with this process, EBM also estimated the stage of disease progression for each participant. The demographic distribution of the two groups was shown in Fig. 4(C). The stage of patients in the T2DM group is higher than that in the HC group (T2DM: 0.54 ± 0.12, HC: 0.49 ± 0.10, *P* = 0.001).

### Sequence of GMV in the T2DM group

Figure 3(D-E) shows a visualization of the probabilistic sequence of abnormality in imaging biomarkers of 35 ROIs (17 ROI on the left and right sides and vermis of the cerebellum) as estimated by EBM. In summary, the GMV in the insula gyrus showed abnormalities first, followed by the GMV in most areas of the deep gray matter nuclei, then the GMV in the temporal gyrus, and finally the GMV in globus pallidus. The disease progression stage, as indicated by the EBM staging, is more advanced in the T2DM group compared to the HC group (T2DM: 0.54 ± 0.12, HC: 0.48 ± 0.10, *P* = 0.001). Details were shown in Fig. 3(F) and Supplementary Fig S1.

### Cognitive function and GMV between two groups

After controlling for age, sex and years of education, covariance analysis was performed. For cognitive function, T2DM group showed significantly lower CVLT-PC1 (T2DM: −0.53 ± 1.96, HC: 0.75 ± 1.96, *P*_FDR_=0.029), CVLT-PC2 (T2DM: −0.84 ± 3.20, HC: 1.22 ± 3.17, *P*_FDR_=0.029), CVLT-PC4 (T2DM: −0.36 ± 1.80, HC: 0.56 ± 2.07, *P*_FDR_=0.029), CVLT-PC (T2DM: −0.48 ± 1.88, HC: 0.68 ± 1.92, *P*_FDR_=0.029) and MoCA (T2DM: 25.09 ± 2.09, HC: 26.23 ± 2.68, *P*_FDR_=0.049) compared with HC group. No significant changes were found in all GMV in all ROIs between these two groups. The results were shown in Table S2 and Fig. 4.

### The association between cognition and clinical risk factors in the T2DM group

**Fig. 5:**
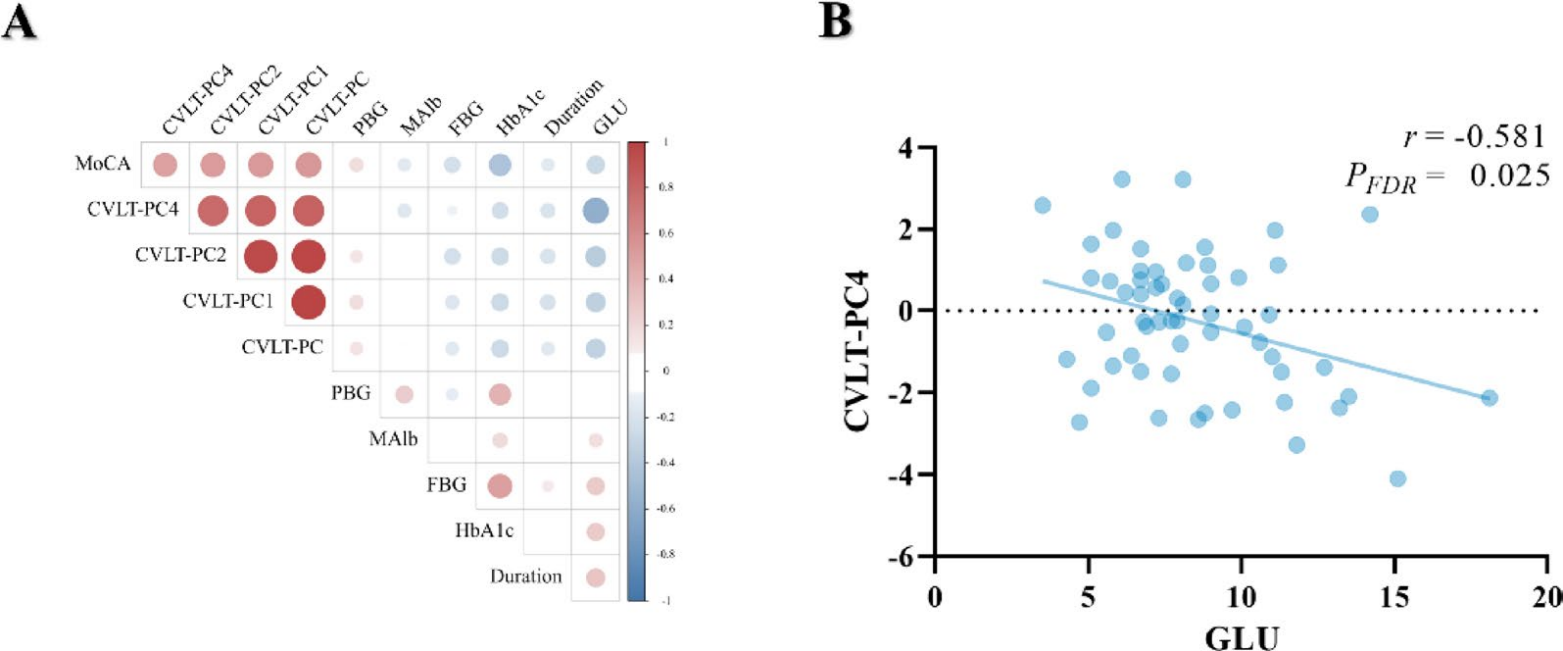
Correlation analysis between abnormal cognitive function and clinical indicators in T2DM group.** (A)** The overall parietal correlation analysis between abnormal cognitive function and clinical indicators in T2DM group. **(B)** The correlations remained significant after FDR correction. MoCA: Montreal Cognitive Assessment; CVLT, California Verbal Learning Test; PC: principal component; PBG: postprandial blood glucose; MAlb: Microalbumin; FBG: fasting blood glucose; GLU: Glucose; HbA1c: hemoglobin A1C; FDR: false discovery rate; T2DM: type 2 diabetes mellitus

Partial correlation analysis was performed between the cognitive scores with significant differences between the two groups and the relevant clinical indicators of T2DM (PBG, FBG, GLU, Malb, duration of illness). After adjusted by FDR, the CVLT-PC4 were negatively associated with random blood glucose (*r* = −0.581, *P*_FDR_ = 0.025). The detailed information was presented in Fig. 5.

## Discussion

This study integrated PCA and EBM to elucidate the temporal progression of cognitive and neuroanatomical alterations in T2DM patients using cross-sectional data. Our findings revealed that cognitive decline in T2DM patients is characterized by an initial deterioration in working memory, followed by progressive impairments in executive function and episodic memory. Neuroimaging analyses further indicated that GMV reductions first manifest in the insula, with subsequent involvement of deep gray matter nuclei and temporal regions. These sequential changes exhibit a strong association with disease progression, offering novel perspectives on the spatiotemporal dynamics of cognitive dysfunction in T2DM.

The application of PCA in this study provided a reliable and efficient approach for dimensionality reduction in the multidimensional assessment of cognitive function in T2DM patients. By employing PCA, we simplified the complex cognitive scores from CVLT, STROOP, and WCST into a few interpretable PCs, each reflecting key cognitive domains such as memory, executive function, and attention. For instance, the PCs derived from CVLT captured memory-related features, including immediate recall and delayed recognition, while those from STROOP and WCST characterized executive functions such as inhibitory control, cognitive flexibility, and abstract thinking. This dimensionality reduction not only minimized data redundancy but also highlighted the core dimensions of cognitive impairment in T2DM, thereby providing a robust analytical framework for subsequent investigations. Previous studies have demonstrated that PCA effectively extracts key variability from neuropsychological test data, exhibiting robust reliability and validity in studies of neurodegenerative disorders such as Alzheimer’s disease, Obstructive Sleep Apnea and common psychiatric illness [8, 18, 44]. In the current study, the Kaiser-Meyer-Olkin measure and Bartlett’s test of sphericity confirmed the suitability of the data for dimensionality reduction, further validating the applicability of PCA in T2DM-related cognitive research. Moreover, the PCs extracted by PCA demonstrated clear clinical interpretability, offering valuable insights into the heterogeneity of cognitive impairment in T2DM patients [26].

The EBM is a statistical framework that evaluates the compatibility between observed and target variables by defining an energy function, demonstrating particular advantages in mapping disease progression patterns through gradient-based minimization techniques [16–18]. In this study, the application of EBM has revealed the spatiotemporal progression of cognitive dysfunction and brain structural abnormalities in T2DM patients. At the cognitive level, the TMT-A scores, which primarily reflect attention and working memory [35, 57], were initially affected, followed by impairments in executive functions and episodic memory. This finding aligns with prior studies that have also identified early deficits in working memory among T2DM patients [22, 45]. Working memory is a central component of short-term memory and its proper functioning depends on the coordinated interaction between the prefrontal cortex and limbic structures such as the hippocampus [14]. The maintenance of this memory is the result of the interaction of long-term memory representations with fundamental cognitive processes, including attention [9, 10]. This dysfunction in T2DM patients may be affected by chronic hyperglycemia and insulin resistance, as these metabolic disturbances may disrupt the prefrontal-hippocampal pathway thus leading to early deficits in working memory [13, 46]. In contrast to the findings of other studies [1, 41], our research indicates that executive dysfunction showed up later than working memory dysfunction in T2DM patients. This implies that cognitive impairment in T2DM patients may be selective in the early stage, and the executive dysfunction may become more pronounced as the disease progresses. Structurally, while no significant differences in GMV were found between T2DM patients and HC at the ROI level after adjusting for covariates, GM atrophy initially appeared in the insular cortex and progressively spread to deep gray matter nuclei and the temporal lobes in EBM analysis. This discrepancy may be attributable to the fact that EBM is capable of detecting sub-threshold effects that are not readily apparent in group-level comparisons. Future studies may consider combining EBM with traditional statistical methods to gain a more comprehensive understanding of neuroanatomical changes in T2DM. Additionally, the insula gyrus, commonly regarded as the integration hub of the brain, is anatomically located between the frontal and temporal lobes, along with the limbic system [38]. This gyrus plays a crucial role in processes such as visceral perception, emotional regulation, and cognitive control [20, 63]. In the early stages of T2DM patients, atrophy of the insula gyrus may disrupt synergistic interactions between memory-related networks (e.g., the hippocampus-ventral prefrontal cortex-insula circuits in the default mode network). Such structural alterations have an adverse effect on cognitive function through impaired information prioritization and contextual memory consolidation [24, 36, 55]. This also provides an important clue to the potential imaging mechanisms underlying the early onset of working memory impairment in T2DM patients. Future studies can further explore the specific relationship between memory alterations and the insular gyrus in T2DM patients to reveal its neurobiological significance in the early stages of the disease.

In our study, T2DM patients exhibited significantly lower MoCA scores compared to the HC group, which is consistent with prior research [1, 46]. The MoCA test, as a comprehensive cognitive screening tool, is sensitive to the subtle cognitive deficits often observed in T2DM patients [6, 11]. Our results further highlight that cognitive impairment is a common and significant complication in T2DM patients. Moreover, specialized tests such as the CVLT and Stroop tests offer deeper insights into specific cognitive domains [32]. In our study, the significant decrease in CVLT-related PCs (PC1, PC2, PC4) indicates that memory dysfunction in T2DM patients has multidimensional characteristics, particularly evident in immediate recall (PC1), delayed retrieval (PC2), and recognition specificity (PC4). These findings are consistent with previous research [42, 46] suggesting that T2DM associated metabolic disturbances may preferentially affect memory encoding and consolidation processes dependent on the hippocampal-prefrontal pathway [15, 23, 27].

Despite significant cognitive impairments, no significant differences in GMV were observed at the ROI level between groups, and these differences may be explained as follows. (1) early functional abnormalities: brain functional abnormalities in the early stages of T2DM, such as reduced neural activity synchronization or changes in white matter microstructure, may precede macrostructural changes [7, 60]; (2) limitations of ROI classification: the existing ROI classification currently might not be able to capture the region-specific atrophy patterns unique to T2DM patients. Previous studies have identified GMV alterations in T2DM patients, particularly in regions such as the temporal gyrus, frontal gyrus, and cerebellum [53, 61, 62]. However, our EBM analysis revealed that the insula gyrus may be the earliest region to exhibit GMV reduction in T2DM patients. This discrepancy could be due to differences in the cognitive status of T2DM patients, potential demographic differences, and different analysis methods. In the future, research could take surface morphometry or functional connectivity analysis into consideration.

Moreover, our study identified a significant negative correlation between CVLT-PC4 and GLU levels, indicating that acute glucose fluctuations may have a more pronounced impact on long-delay recognition memory than chronic hyperglycemia as measured by HbA1c. This finding suggests that the immediate and transient changes in blood glucose levels, as captured by GLU, may play a more critical role in cognitive dysfunction in T2DM patients compared to the stable glucose levels measured by FBG, PBG, and HbA1c. This discrepancy can be explained by several factors. (1) GLU measurements reflect the immediate blood glucose levels, which can capture acute fluctuations that may directly impact cognitive performance [21, 23]. Acute hyperglycemia can lead to impaired synaptic plasticity and neurogenesis, which are essential for memory encoding and retrieval [49, 56, 59]. These acute changes may directly affect cognitive performance, particularly in long-delay recognition memory (CVLT-PC4 in this study). (2) In contrast, FBG and PBG reflect specific time-point measurements and may not capture the dynamic changes in blood glucose levels throughout the day. (3) As for HbA1c, this indicator, while a marker of long-term glucose control, reflects the average blood glucose levels over the past 2–3 months and may not capture the acute fluctuations that can impact cognitive function [54]. Chronic hyperglycemia, as measured by HbA1c, may contribute to cognitive impairment through chronic metabolic disturbances, but these effects may be more gradual and less pronounced in the short term compared to the immediate effects of acute glucose fluctuations [13, 30]. Future studies should consider the combined effects of both acute and chronic glucose levels to better understand their impact on cognitive decline in T2DM patients.

This study has several limitations. First, the modest sample size may limit the generalizability of our findings, and the geographic specificity of our sample may not be representative of broader T2DM populations. Future studies should include larger, more diverse cohorts to address these issues. Second, the cross-sectional design restricts causal inferences between T2DM and cognitive impairment. Longitudinal studies are needed to validate our observations and explore underlying mechanisms and therapeutic interventions. Third, our focus on GMV provides structural insights but omits functional aspects. Integrating multimodal neuroimaging techniques, such as functional connectivity (neuronal function) and arterial spin labeling (cerebral blood), could offer a more comprehensive understanding. Finally, we could not combine cognitive and neuroimaging markers in the EBM model due to the lack of significant GMV differences and model limitations with numerous features. We hope future research addresses these gaps to enhance our understanding of cognitive dysfunction in T2DM.

## Conclusion

In conclusion, this study delineates a sequential pattern of cognitive decline in T2DM, starting from attentional and working memory deficits, followed by executive dysfunction and episodic memory impairment. Concurrently, hierarchical GMV reduction was observed, initiating in the insular cortex and progressing to deep gray nuclei. Notably, long-delay recognition memory showed increased vulnerability with elevated GLU levels. These findings suggest that GMV loss in the insular gyrus and memory decline may serve as potential biomarkers for monitoring T2DM-associated cognitive impairment. Our results emphasize the need for dynamic metabolic-cognitive surveillance in clinical practice to better manage and mitigate cognitive decline in T2DM patients.

## Supplementary Information

Supplementary Material 1

## Data Availability

Upon reasonable request, the original data set can be obtained from the corresponding author.

## Declarations

Written informed consent was obtained from all participants.

## References

[1] Antal B, McMahon LP, Sultan SF, et al. Type 2 diabetes mellitus accelerates brain aging and cognitive decline: complementary findings from UK Biobank and meta-analyses. Elife. 2022. 10.7554/eLife.73138. 35608247 10.7554/eLife.73138 PMC9132576

[2] Banquet-Teran J, Johnson-Restrepo B, Hernandez-Morelo A, et al. Linear and nonlinear calibration methods for predicting mechanical properties of polypropylene pellets using Raman spectroscopy. Appl Spectrosc. 2016;70(7):1118–27. 10.1177/0003702816652322. 27287847 10.1177/0003702816652322

[3] Biessels GJ, Despa F. Cognitive decline and dementia in diabetes mellitus: mechanisms and clinical implications. Nat Rev Endocrinol. 2018;14(10):591–604. 10.1038/s41574-018-0048-7. 30022099 10.1038/s41574-018-0048-7 PMC6397437

[4] Callisaya ML, Beare R, Moran C, et al. Type 2 diabetes mellitus, brain atrophy and cognitive decline in older people: a longitudinal study. Diabetologia. 2019;62(3):448–58. 10.1007/s00125-018-4778-9. 30547230 10.1007/s00125-018-4778-9

[5] Chen B, Zhou H, Liu X, et al. Correlations of gray matter volume with peripheral cytokines in Parkinson’s disease. Neurobiol Dis. 2024;201:106693. 10.1016/j.nbd.2024.106693. 39368669 10.1016/j.nbd.2024.106693

[6] Chen T, Liu YL, Li F, et al. Association of waist-to-hip ratio adjusted for body mass index with cognitive impairment in middle-aged and elderly patients with type 2 diabetes mellitus: a cross-sectional study. BMC Public Health. 2024;24(1):2424. 10.1186/s12889-024-19985-7. 39243030 10.1186/s12889-024-19985-7 PMC11378611

[7] Cheng P, Song S, Li Y, et al. Aberrant functional connectivity of the posterior cingulate cortex in type 2 diabetes without cognitive impairment and microvascular complications. Front Endocrinol (Lausanne). 2021;12:722861. 10.3389/fendo.2021.722861. 34759889 10.3389/fendo.2021.722861 PMC8573207

[8] Chopra S, Dhamala E, Lawhead C, et al. Generalizable and replicable brain-based predictions of cognitive functioning across common psychiatric illness. Sci Adv. 2024;10(45):eadn1862. 10.1126/sciadv.adn1862. 39504381 10.1126/sciadv.adn1862 PMC11540040

[9] Daume J, Kaminski J, Salimpour Y, et al. Persistent activity during working memory maintenance predicts long-term memory formation in the human hippocampus. Neuron. 2024;112(23):3957–e39683. 10.1016/j.neuron.2024.09.013. 39406238 10.1016/j.neuron.2024.09.013 PMC11624075

[10] Daume J, Kaminski J, Schjetnan AGP, et al. Control of working memory by phase-amplitude coupling of human hippocampal neurons. Nature. 2024;629(8011):393–401. 10.1038/s41586-024-07309-z. 38632400 10.1038/s41586-024-07309-z PMC11078732

[11] Ding J, Zou L, Xu R, et al. The research of Dapagliflozin on cognitive function in middle-aged and older patients with type 2 diabetes mellitus and mild cognitive impairment: a 36-week prospective parallel control study. Eur J Pharmacol. 2025;1002:177819. 10.1016/j.ejphar.2025.177819. 40490172 10.1016/j.ejphar.2025.177819

[12] Dong Y, Wang Q, Yao H, et al. A promising structural magnetic resonance imaging assessment in patients with preclinical cognitive decline and diabetes mellitus. J Cell Physiol. 2019;234(10):16838–46. 10.1002/jcp.28359. 30786010 10.1002/jcp.28359

[13] Ehtewish H, Arredouani A, El-Agnaf O. Diagnostic, prognostic, and mechanistic biomarkers of diabetes mellitus-associated cognitive decline. Int J Mol Sci. 2022. 10.3390/ijms23116144. 35682821 10.3390/ijms23116144 PMC9181591

[14] Eriksson J, Vogel EK, Lansner A, et al. Neurocognitive Archit Working Memory Neuron. 2015;88(1):33–46. 10.1016/j.neuron.2015.09.020. 10.1016/j.neuron.2015.09.020 PMC4605545 26447571

[15] Ertas B, Hazar-Yavuz AN, Topal F, et al. *Rosa canina* L. improves learning and memory-associated cognitive impairment by regulating glucose levels and reducing hippocampal insulin resistance in high-fat diet/streptozotocin-induced diabetic rats. J Ethnopharmacol. 2023;313:116541. 10.1016/j.jep.2023.116541. 37088237 10.1016/j.jep.2023.116541

[16] Eshaghi A, Marinescu RV, Young AL, et al. Progression of regional grey matter atrophy in multiple sclerosis. Brain. 2018;141(6):1665–77. 10.1093/brain/awy088. 29741648 10.1093/brain/awy088 PMC5995197

[17] Eshaghi A, Young AL, Wijeratne PA, et al. Identifying multiple sclerosis subtypes using unsupervised machine learning and MRI data. Nat Commun. 2021;12(1):2078. 10.1038/s41467-021-22265-2. 33824310 10.1038/s41467-021-22265-2 PMC8024377

[18] Firth NC, Primativo S, Brotherhood E, et al. Sequences of cognitive decline in typical Alzheimer’s disease and posterior cortical atrophy estimated using a novel event-based model of disease progression. Alzheimers Dement. 2020;16(7):965–73. 10.1002/alz.12083. 32489019 10.1002/alz.12083 PMC8432168

[19] Fonteijn HM, Modat M, Clarkson MJ, et al. An event-based model for disease progression and its application in Familial Alzheimer’s disease and Huntington’s disease. Neuroimage. 2012;60(3):1880–9. 10.1016/j.neuroimage.2012.01.062. 22281676 10.1016/j.neuroimage.2012.01.062

[20] Gasquoine PG. Contributions of the insula to cognition and emotion. Neuropsychol Rev. 2014;24(2):77–87. 10.1007/s11065-014-9246-9. 24442602 10.1007/s11065-014-9246-9

[21] Goncalves JS, Seica RM, Laranjinha J, et al. Impairment of neurovascular coupling in the hippocampus due to decreased nitric oxide bioavailability supports early cognitive dysfunction in type 2 diabetic rats. Free Radic Biol Med. 2022;193(Pt 2):669–75. 10.1016/j.freeradbiomed.2022.11.009. 36372286 10.1016/j.freeradbiomed.2022.11.009

[22] Gorniak SL, Lu FY, Lee BC, et al. Cognitive impairment and postural control deficit in adults with type 2 diabetes. Diabetes Metab Res Rev. 2019;35(2):e3089. 10.1002/dmrr.3089. 30338902 10.1002/dmrr.3089 PMC6590678

[23] Gupta M, Pandey S, Rumman M, et al. Molecular mechanisms underlying hyperglycemia associated cognitive decline. IBRO Neurosci Rep. 2023;14:57–63. 10.1016/j.ibneur.2022.12.006. 36590246 10.1016/j.ibneur.2022.12.006 PMC9800261

[24] He Z, Li S, Mo L, et al. The VLPFC-engaged voluntary emotion regulation: combined TMS-fMRI evidence for the neural circuit of cognitive reappraisal. J Neurosci. 2023;43(34):6046–60. 10.1523/JNEUROSCI.1337-22.2023. 37507228 10.1523/JNEUROSCI.1337-22.2023 PMC10451149

[25] Hu B, Wang X, He JB, et al. Structural and functional brain changes in perimenopausal women who are susceptible to migraine: a study protocol of multi-modal MRI trial. BMC Med Imaging. 2018;18(1):26. 10.1186/s12880-018-0272-6. 30189858 10.1186/s12880-018-0272-6 PMC6127929

[26] Hu B, Yu Y, Yu XW, et al. Sequence of episodic memory-related behavioral and brain-imaging abnormalities in type 2 diabetes. Nutr Diabetes. 2025;15(1):1. 10.1038/s41387-025-00359-w. 39893169 10.1038/s41387-025-00359-w PMC11787324

[27] Huang RR, Jia BH, Xie L, et al. Spatial working memory impairment in primary onset middle-age type 2 diabetes mellitus: an ethology and BOLD-fMRI study. J Magn Reson Imaging. 2016;43(1):75–87. 10.1002/jmri.24967. 26094886 10.1002/jmri.24967

[28] Jiang Y, Luo C, Wang J, et al. Neurostructural subgroup in 4291 individuals with schizophrenia identified using the subtype and stage inference algorithm. Nat Commun. 2024;15(1):5996. 10.1038/s41467-024-50267-3. 39013848 10.1038/s41467-024-50267-3 PMC11252381

[29] Jiang Y, Wang J, Zhou E, et al. Neuroimaging biomarkers define neurophysiological subtypes with distinct trajectories in schizophrenia. Nat Ment Health. 2023;1(3):186–99. 10.1038/s44220-023-00024-0.

[30] Kielstein JT. Glucose levels and risk of dementia. N Engl J Med. 2013;369(19):1863. 10.1056/NEJMc1311765. 24195564 10.1056/NEJMc1311765

[31] Kim HE, Kim JJ, Seok JH, et al. Resting-state functional connectivity and cognitive performance in aging adults with cognitive decline: a data-driven multivariate pattern analysis. Compr Psychiatry. 2024;129:152445. 10.1016/j.comppsych.2023.152445. 38154288 10.1016/j.comppsych.2023.152445

[32] Kim RY, Joo Y, Ha E, et al. Alterations in brain morphometric networks and their relationship with memory dysfunction in patients with type 2 diabetes mellitus. Exp Neurobiol. 2024;33(2):107–17. 10.5607/en24005. 38724480 10.5607/en24005 PMC11089400

[33] Leuzy A, Smith R, Cullen NC, et al. Biomarker-based prediction of longitudinal tau positron emission tomography in Alzheimer disease. JAMA Neurol. 2022;79(2):149–58. 10.1001/jamaneurol.2021.4654. 34928318 10.1001/jamaneurol.2021.4654 PMC8689441

[34] Li M, Li Y, Liu Y, et al. Altered hippocampal subfields volumes is associated with memory function in type 2 diabetes mellitus. Front Neurol. 2021;12:756500. 10.3389/fneur.2021.756500. 34899576 10.3389/fneur.2021.756500 PMC8657943

[35] Liu Y, Xu Y, Tong S. Serum glial cell line-derived neurotrophic factor: a potential biomarker for white matter alteration in Parkinson’s disease with mild cognitive impairment. Front Neurosci. 2024;18:1370787. 10.3389/fnins.2024.1370787. 39513043 10.3389/fnins.2024.1370787 PMC11541347

[36] Llorens A, Bellier L, Blenkmann AO, et al. Decision and response monitoring during working memory are sequentially represented in the human insula. iScience. 2023;26(10):107653. 10.1016/j.isci.2023.107653. 37674986 10.1016/j.isci.2023.107653 PMC10477069

[37] Milne NT, Bucks RS, Davis WA, et al. Hippocampal atrophy, asymmetry, and cognition in type 2 diabetes mellitus. Brain Behav. 2018;8(1):e00741. 10.1002/brb3.741. 29568674 10.1002/brb3.741 PMC5853633

[38] Namkung H, Kim SH, Sawa A. The insula: an underestimated brain area in clinical neuroscience, psychiatry, and neurology. Trends Neurosci. 2017;40(4):200–7. 10.1016/j.tins.2017.02.002. 28314446 10.1016/j.tins.2017.02.002 PMC5538352

[39] Ni MH, Yu Y, Yang Y, et al. Functional-structural decoupling in visual network is associated with cognitive decline in patients with type 2 diabetes mellitus: evidence from a multimodal MRI analysis. Brain Imaging Behav. 2024;18(1):73–82. 10.1007/s11682-023-00801-6. 37874444 10.1007/s11682-023-00801-6

[40] Oxtoby NP, Young AL, Cash DM, et al. Data-driven models of dominantly-inherited Alzheimer’s disease progression. Brain. 2018;141(5):1529–44. 10.1093/brain/awy050. 29579160 10.1093/brain/awy050 PMC5920320

[41] Palta P, Carlson MC, Crum RM, et al. Diabetes and cognitive decline in older adults: the Ginkgo evaluation of memory study. The Journals of Gerontology: Series A. 2017;73(1):123–30. 10.1093/gerona/glx076. 10.1093/gerona/glx076 PMC5861864 28510619

[42] Palta P, Schneider ALC, Biessels GJ, et al. Magnitude of cognitive dysfunction in adults with type 2 diabetes: a meta-analysis of six cognitive domains and the most frequently reported neuropsychological tests within domains. J Int Neuropsychol Soc. 2014;20(3):278–91. 10.1017/S1355617713001483. 24555960 10.1017/S1355617713001483 PMC4132660

[43] Pascuzzo R, Oxtoby NP, Young AL, et al. Prion propagation estimated from brain diffusion MRI is subtype dependent in sporadic Creutzfeldt-Jakob disease. Acta Neuropathol. 2020;140(2):169–81. 10.1007/s00401-020-02168-0. 32535770 10.1007/s00401-020-02168-0 PMC7360647

[44] Pase MP, Harrison S, Misialek JR, et al. Sleep architecture, obstructive sleep apnea, and cognitive function in adults. JAMA Netw Open. 2023;6(7):e2325152. 10.1001/jamanetworkopen.2023.25152. 37462968 10.1001/jamanetworkopen.2023.25152 PMC10354680

[45] Sadanand S, Balachandar R, Bharath S. Memory and executive functions in persons with type 2 diabetes: a meta-analysis. Diabetes Metab Res Rev. 2016;32(2):132–42. 10.1002/dmrr.2664. 25963303 10.1002/dmrr.2664

[46] Srikanth V, Sinclair AJ, Hill-Briggs F, et al. Type 2 diabetes and cognitive dysfunction-towards effective management of both comorbidities. Lancet Diabetes Endocrinol. 2020;8(6):535–45. 10.1016/S2213-8587(20)30118-2. 32445740 10.1016/S2213-8587(20)30118-2

[47] Sun H, Saeedi P, Karuranga S, et al. IDF diabetes atlas: global, regional and country-level diabetes prevalence estimates for 2021 and projections for 2045. Diabetes Res Clin Pract. 2022;183:109119. 10.1016/j.diabres.2021.109119. 34879977 10.1016/j.diabres.2021.109119 PMC11057359

[48] Tan S, Zhou C, Wen J, et al. Presence but not the timing of onset of REM sleep behavior disorder distinguishes evolution patterns in Parkinson’s disease. Neurobiol Dis. 2023;180:106084. 10.1016/j.nbd.2023.106084. 36931531 10.1016/j.nbd.2023.106084

[49] van Duinkerken E, Ryan CM. Diabetes mellitus in the young and the old: effects on cognitive functioning across the life span. Neurobiol Dis. 2020;134:104608. 10.1016/j.nbd.2019.104608. 31494283 10.1016/j.nbd.2019.104608

[50] Venkatraghavan V, Bron EE, Niessen WJ, et al. Disease progression timeline estimation for Alzheimer’s disease using discriminative event based modeling. Neuroimage. 2019;186:518–32. 10.1016/j.neuroimage.2018.11.024. 30471388 10.1016/j.neuroimage.2018.11.024

[51] Venkatraghavan V, Pascuzzo R, Bron EE, et al. A discriminative event-based model for subtype diagnosis of sporadic Creutzfeldt-Jakob disease using brain MRI. Alzheimers Dement. 2023;19(8):3261–71. 10.1002/alz.12939. 36749840 10.1002/alz.12939

[52] Wang S, Nie F, Wang Z, et al. Robust principal component analysis via joint reconstruction and projection. IEEE Trans Neural Netw Learn Syst. 2024;35(5):7175–89. 10.1109/TNNLS.2022.3214307. 36367910 10.1109/TNNLS.2022.3214307

[53] Wu G, Lin L, Zhang Q, et al. Brain gray matter changes in type 2 diabetes mellitus: a meta-analysis of whole-brain voxel-based morphometry study. J Diabetes Complications. 2017;31(12):1698–703. 10.1016/j.jdiacomp.2017.09.001. 29033311 10.1016/j.jdiacomp.2017.09.001

[54] Xia W, Luo Y, Chen YC, et al. Glucose fluctuations are linked to disrupted brain functional architecture and cognitive impairment. J Alzheimers Dis. 2020;74(2):603–13. 10.3233/JAD-191217. 32065795 10.3233/JAD-191217

[55] Yang W, Jia H, Feng Q, et al. Functional connectivity between right-lateralized ventrolateral prefrontal cortex and insula mediates reappraisal’s link to memory control. J Affect Disord. 2021;290:316–23. 10.1016/j.jad.2021.04.057. 34020206 10.1016/j.jad.2021.04.057

[56] Yang W, Si SC, Li J, et al. Nlrp3 inhibitor alleviates glycemic variability-induced cognitive impairment in aged rats with type 2 diabetes mellitus. Mol Cell Endocrinol. 2025;595:112406. 10.1016/j.mce.2024.112406. 39489213 10.1016/j.mce.2024.112406

[57] Yoshimura K, Shima A, Kambe D, et al. Neural substrates underlying distinct dual cognitive syndromes in parkinson’s disease. Eur J Neurol. 2025;32(1):e70022. 10.1111/ene.70022. 39716907 10.1111/ene.70022 PMC11666957

[58] Young AL, Oxtoby NP, Daga P, et al. A data-driven model of biomarker changes in sporadic Alzheimer’s disease. Brain. 2014;137(Pt 9):2564–77. 10.1093/brain/awu176. 25012224 10.1093/brain/awu176 PMC4132648

[59] Yu Y, Yan LF, Sun Q, et al. Neurovascular decoupling in type 2 diabetes mellitus without mild cognitive impairment: potential biomarker for early cognitive impairment. Neuroimage. 2019;200:644–58. 10.1016/j.neuroimage.2019.06.058. 31252056 10.1016/j.neuroimage.2019.06.058

[60] Zhang D, Huang Y, Liu S, et al. Structural and functional connectivity alteration patterns of the cingulate gyrus in type 2 diabetes. Ann Clin Transl Neurol. 2023;10(12):2305–15. 10.1002/acn3.51918. 37822294 10.1002/acn3.51918 PMC10723245

[61] Zhang D, Lei Y, Gao J, et al. Right frontoinsular cortex: a potential imaging biomarker to evaluate T2DM-induced cognitive impairment. Front Aging Neurosci. 2021;13:674288. 10.3389/fnagi.2021.674288. 34122050 10.3389/fnagi.2021.674288 PMC8193040

[62] Zhang HY, Shen G, Yang C, et al. The reduced gray matter volume and functional connectivity of the cerebellum in type 2 diabetes mellitus with high insulin resistance. Neuroendocrinology. 2024;114(4):386–99. 10.1159/000535860. 38113872 10.1159/000535860

[63] Zhang R, Deng H, Xiao X. The insular cortex: an interface between sensation, emotion and cognition. Neurosci Bull. 2024;40(11):1763–73. 10.1007/s12264-024-01211-4. 38722464 10.1007/s12264-024-01211-4 PMC11607240

[64] Zheng Y, Ley SH, Hu FB. Global aetiology and epidemiology of type 2 diabetes mellitus and its complications. Nat Rev Endocrinol. 2018;14(2):88–98. 10.1038/nrendo.2017.151. 29219149 10.1038/nrendo.2017.151

