# Supplementary material for: Temporal dynamic of cognitive decline in type 2 diabetes mellitus patients: a multimodal biomarker analysis using event-based modal and principal component analysis
Source: Diabetol Metab Syndr. 2025 Nov 14;17:429. doi: 10.1186/s13098-025-02003-0 (PMC12619481; doi:10.1186/s13098-025-02003-0)
Supplement: Supplementary file 1 — Supplementary Material 1 [file 13098_2025_2003_MOESM1_ESM.docx]

- **Baseline cognitive status**

All participants underwent comprehensive neuropsychological assessment at enrollment, including MMSE, MoCA, and domain-specific tests (CVLT, WCST, Stroop, TMT). However, no clinical diagnosis of cognitive impairment (e.g., mild cognitive impairment (MCI) or subjective cognitive decline (SCD)) was made at baseline, and no cognitive status-based exclusion was applied. Instead, we retained the full spectrum of cognitive performance in T2DM patients to ensure heterogeneity required for event-based modeling. For descriptive purposes only, we retrospectively estimated cognitive status using MMSE and MoCA cutoffs commonly used in population-based studies:

- - MMSE ≥ 27 and MoCA ≥ 26: likely cognitively unimpaired;
  - MMSE < 27 or MoCA < 26: possible cognitive impairment (not clinically confirmed).

These labels were not used for inclusion/exclusion, but only to characterize the sample. No participant had a history of dementia, stroke, epilepsy, or other neurological disorders that could confound cognitive assessment.

**Table S1** Cognitive Profile Based on MMSE and MoCA Cut-offs of T2DM group and HC group

| Baseline Cognitive Performance | T2DM (n=119) | HC (n=87) |
| --- | --- | --- |
| MMSE ≥ 27 and MoCA ≥ 26 | 56 | 66 |
| MMSE < 27 or MoCA < 26 | 63 | 21 |

- **CAT12 Quality Scoring**

During the preprocessing phase, all T1-weighted images underwent segmentation and normalization using the Computational Anatomy Toolbox (CAT12). As part of its automated segmentation procedure, CAT12 assigns a quality score to each image, evaluating factors such as signal-to-noise ratio, image artifacts, contrast, and the precision of tissue classification. This score is essential for assessing the reliability of the subsequent gray matter volume analysis. To guarantee the quality of our results, only images with a CAT12 quality score of 'C' or above were included in the statistical analysis.

**
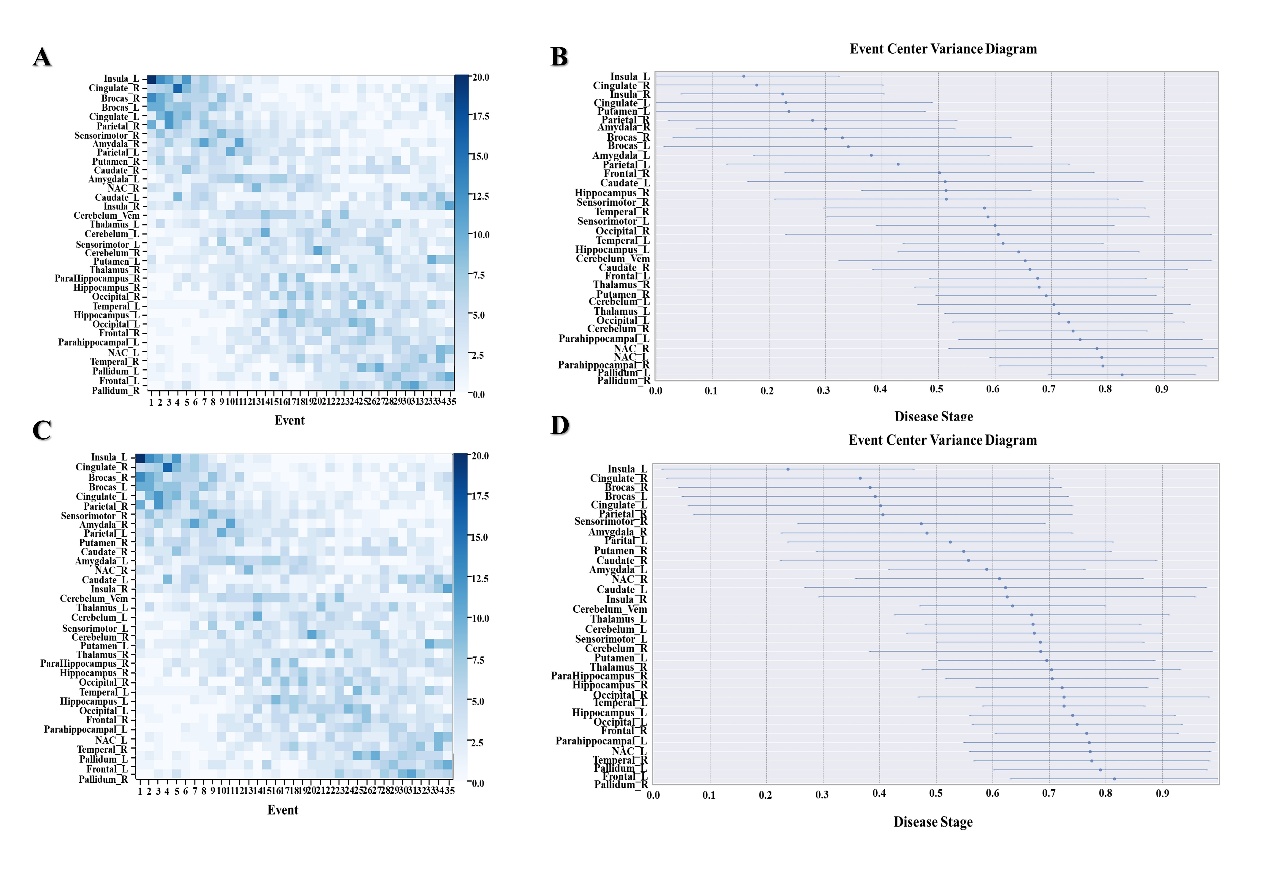
**

**Supplementary Fig S1.** Analysis of brain imaging biomarkers in Type 2 Diabetes Mellitus (T2DM) using the Event-Based Model (EBM).

**(A, B)** These panels illustrate the results without including Total Intracranial Volume (TIV) as a covariate in the EBM analysis. **(A)** shows the frequency of parameter localization during bootstrap resampling, with the saturation gradient of each square indicating this frequency. Maximal chromatic density highlights the predominant temporal sequence for each biomarker in cognition and GMV among the T2DM group. **(B)** presents the Event Center Variance Diagram, which illustrates the estimated stage at which features deviate from normality and the associated variance within the population for cognition and GMV in the T2DM group.

**(C, D)** These panels display the outcomes when TIV is included as a covariate in the EBM analysis. **(C)** mirrors (A) but represents the analysis without TIV, showing a similar pattern of temporal sequence for biomarkers. **(D)** corresponds to (B) and provides the Event Center Variance Diagram for the analysis without TIV, indicating the stage of deviation from normality and associated variance.

In both scenarios, with or without TIV as a covariate, the left insula (Insula_L) consistently shows the earliest changes, underscoring the robustness of our findings across different analytical approaches. L: left; R: right; NAC: Nucleus Accumbens.

**Table S2** Cognitive domains tested by our neuropsychological test battery of T2DM group and HC group.

| Cognitive domain | T2DM (n=119) | HC (n=87) | *F* | *P_FDR_* |
| --- | --- | --- | --- | --- |
| **General cognition** |  |  |  |  |
| MoCA | 25.09 ± 2.094 | 26.23 ± 2.684 | 6.919 | 0.049^*^ |
| MMSE | 28.56 ± 1.449 | 28.77 ± 1.230 | -2.461 | 0.345 |
| **Memory** |  |  |  |  |
| zCVLT-PC1 | -0.530± 1.959 | 0.746 ± 1.959 | -8.871 | 0.029^*^ |
| zCVLT-PC2 | -0.843 ± 3.200 | 1.217 ± 3.173 | -8.610 | 0.029^*^ |
| zCVLT-PC3 | 0.565 ± 2.614 | -0.775 ± 2.164 | 0.903 | 0.480 |
| zCVLT-PC4 | -0.356 ± 1.796 | 0.559 ± 2.065 | -8.867 | 0.029^*^ |
| zCVLT-PC | -0.480 ± 1.884 | 0.677 ± 1.918 | -8.748 | 0.029^*^ |
| **Attention and working memory** |  |  |  |  |
| TMT-A | 59.833 ± 31.860 | 51.903 ± 21.899 | 1.162 | 0.480 |
| **Executive function** |  |  |  |  |
| zSTROOP-PC1 | -0.553 ± 2.101 | 0.704 ± 2.788 | -0.426 | 0.547 |
| zSTROOP-PC2 | -0.514 ± 2.574 | 0.654 ± 3.821 | -0.883 | 0.480 |
| zSTROOP-PC | -0.547 ± 2.127 | 0.697 ± 2.910 | -0.480 | 0.547 |
| zWCST-PC1 | 0.780 ± 3.203 | -1.015 ± 2.851 | 1.575 | 0.416 |
| zWCST-PC2 | 0.035 ± 1.343 | -0.041 ± 2.122 | 2.630 | 0.345 |
| zWCST-PC3 | 0.250 ± 1.664 | -0.338 ± 1.400 | 2.154 | 0.361 |
| zWCST-PC4 | 0.077 ± 1.664 | -0.099 ± 1.572 | 0.171 | 0.682 |
| zWCST-PC | 0.538 ± 2.149 | -0.700 ± 2.081 | 0.657 | 0.502 |
| TMT-B | 164.928 ± 95.939 | 141.235 ± 99.983 | 0.707 | 0.502 |
| **General mental status** |  |  |  |  |
| SAS | 42.92 ± 7.349 | 39.83 ± 7.269 | 0.973 | 0.480 |
| SDS | 47.65 ± 7.745 | 43.36 ± 9.788 | 1.614 | 0.416 |

Data were reported as mean ± SD. z represents the result after principal component analysis. T2DM, type 2 diabetes mellitus; HC, healthy controls; MMSE, Mini-Mental State Examination; MoCA, Montreal Cognitive Assessment; CVLT, California Verbal Learning Test; TMT: Trail Making Test; STROOP, Stroop Color Word Test; WCST, Wisconsin Card Sorting Test; PC: principal component; SAS: Self-Rating Anxiety Scale; SDS: Self-rating depression scale; FDR: false discovery rate. ^*^*P* < 0.05.

**Table S3** Image feature of the T2DM group and HC group.

| Brain Area | T2DM (n=111) | HC (n=83) | *F* | *P_FDR_* |
| --- | --- | --- | --- | --- |
| Hippocampus_L | 0.475 ± 0.048 | 0.485 ± 0.052 | -1.452 | 0.861 |
| Hippocampus_R | 0.455 ± 0.044 | 0.463 ± 0.046 | -1.527 | 0.861 |
| Parahippocampal_L | 0.445 ± 0.043 | 0.461 ± 0.049 | -5.527 | 0.720 |
| Parahippocampal_R | 0.458 ± 0.046 | 0.471 ± 0.047- | -3.138 | 0.861 |
| Amygdala_L | 0.564 ± 0.062 | 0.572 ± 0.060 | -0.705 | 0.861 |
| Amygdala_R | 0.536 ± 0.055 | 0.543 ± 0.053 | -0.199 | 0.861 |
| Caudate_L | 0.471 ± 0.054 | 0.473 ± 0.060 | -0.024 | 0.878 |
| Caudate_R | 0.434 ± 0.051 | 0.431 ± 0.056 | 0.505 | 0.861 |
| Putamen_L | 0.509 ± 0.062 | 0.515 ± 0.051 | -0.130 | 0.861 |
| Putamen_R | 0.458 ± 0.055 | 0.461 ± 0.051 | -0.085 | 0.861 |
| Pallidum_L | 0.163 ± 0.034 | 0.168 ± 0.040 | 0.205 | 0.861 |
| Pallidum_R | 0.181 ± 0.035 | 0.186 ± 0.039 | -0.104 | 0.861 |
| Thalamus_L | 0.477 ± 0.044 | 0.476 ± 0.046 | 0.203 | 0.861 |
| Thalamus_R | 0.510 ± 0.048 | 0.510 ± 0.053 | -0.056 | 0.861 |
| Cingulate_L | 0.586 ± 0.059 | 0.593 ± 0.056 | -0.429 | 0.861 |
| Cingulate_R | 0.594 ± 0.064 | 0.599 ± 0.059 | -0.043 | 0.861 |
| Frontal_L | 0.416 ± 0.042 | 0.420 ± 0.046 | -0.475 | 0.861 |
| Frontal_R | 0.392 ± 0.042 | 0.399 ± 0.044 | -0.548 | 0.861 |
| Parietal_L | 0.357 ± 0.035 | 0.359 ± 0.035 | -0.144 | 0.861 |
| Parietal_R | 0.359 ± 0.036 | 0.362 ± 0.035 | -0.299 | 0.861 |
| Temperal_L | 0.337 ± 0.031 | 0.338 ± 0.034 | -0.044 | 0.861 |
| Temperal_R | 0.352 ± 0.031 | 0.356 ± 0.347 | -0.415 | 0.861 |
| Occipital_L | 0.421 ± 0.038 | 0.428 ± 0.042 | -1.308 | 0.861 |
| Occipital_R | 0.413 ± 0.038 | 0.421 ± 0.040 | -1.776 | 0.861 |
| Insula_L | 0.356 ± 0.033 | 0.354 ± 0.036 | 0.474 | 0.861 |
| Insula_R | 0.365 ± 0.036 | 0.361 ± 0.038 | 0.441 | 0.861 |
| Cerebelum_L | 0.471 ± 0.051 | 0.483 ± 0.052 | -2.734 | 0.861 |
| Cerebelum_R | 0.477 ± 0.048 | 0.488 ± 0.049 | -2.511 | 0.861 |
| Cerebelum_Vem | 0.430 ± 0.040 | 0.433 ± 0.042 | -0.286 | 0.861 |
| Sensorimotor_L | 0.427 ± 0.041 | 0.429 ± 0.042 | -0.146 | 0.861 |
| Sensorimotor_R | 0.343 ± 0.033 | 0.343 ± 0.031 | 0.075 | 0.861 |
| Brocas_L | 0.322 ± 0.033 | 0.321 ± 0.032 | 0.150 | 0.861 |
| Brocas_R | 0.321 ± 0.032 | 0.318 ± 0.031 | 0.353 | 0.861 |
| NAC_L | 0.339 ± 0.037 | 0.340 ± 0.041 | -0.071 | 0.861 |
| NAC_R | 0.309 ± 0.035 | 0.311 ± 0.035 | -0.227 | 0.861 |
| TIV | 81.897 ± 9.364 | 82.026 ± 12.666 | -0.160 | 0.861 |

Data were reported as mean ± SD. T2DM, type 2 diabetes mellitus; HC, healthy controls; Left: left; R: right; NAC: Nucleus Accumbens; TIV: total intracranial volume; FDR: false discovery rate.

**Table S4** Correlation analysis (*p* value) between abnormal cognitive function and clinical indicators in T2DM group after false discovery rate.

|  | CVLT-PC1 | CVLT-PC2 | CVLT-PC4 | CVLT-PC | MoCA |
| --- | --- | --- | --- | --- | --- |
| Duration | 0.696 | 0.696 | 0.696 | 0.696 | 0.960 |
| GLU | 0.375 | 0.375 | 0.025 | 0.375 | 0.737 |
| FBG | 0.696 | 0.696 | 0.750 | 0.722 | 0.696 |
| PBG | 0.750 | 0.781 | 0.781 | 0.758 | 0.722 |
| MAlb | 0.717 | 0.722 | 0.696 | 0.696 | 0.696 |
| HbA1c | 0.616 | 0.616 | 0.616 | 0.616 | 0.105 |
